# Supplementary figures and images for: Unlocking ensemble ecosystem modelling for large and complex networks
Source: PLoS Comput Biol. 2024 Mar 14;20(3):e1011976. doi: 10.1371/journal.pcbi.1011976 (PMC10965070; doi:10.1371/journal.pcbi.1011976)

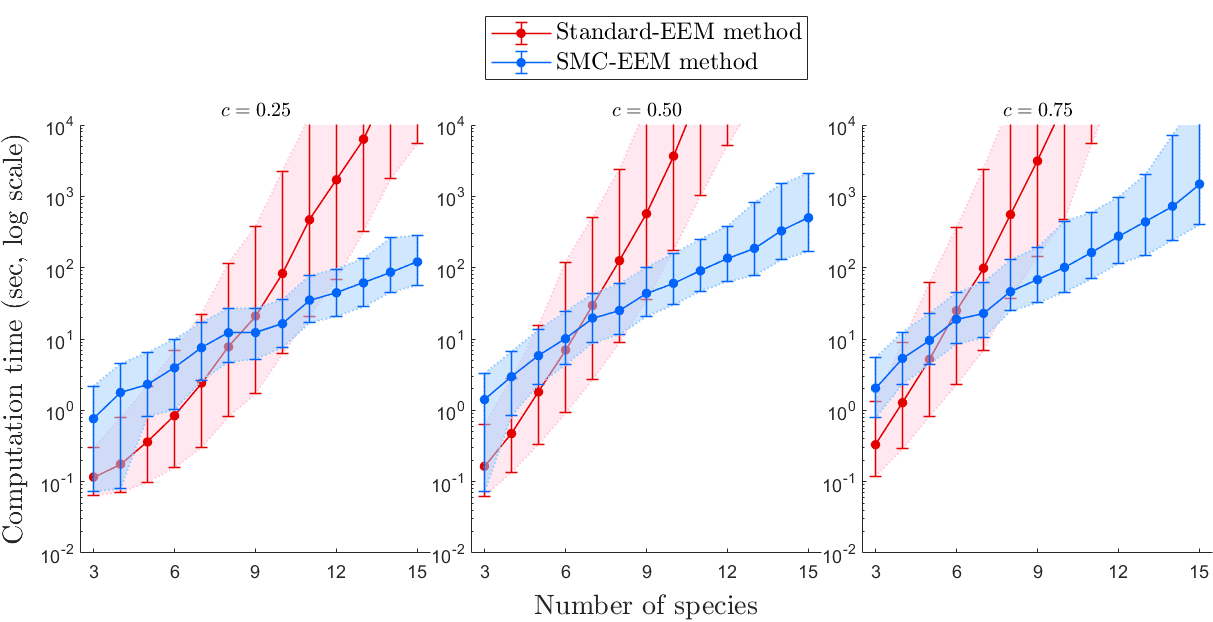

Supplement: S1 Fig — The computation time needed to generate an ensemble of 1000 feasible and stable ecosystem models using a connectance probability of c = 0.25 (left), c = 0.5 (middle) and c = 0.75 (right), for both the standard-EEM and SMC-EEM methods. This figure shows the medians (dots) and 7.5–92.5% quantiles (error bars) of computation times for producing the results. Note, the computation time for any one ecosystem network was capped at 104 seconds due to the computational burden of the simulation study. More densely connected ecosystems (higher value of c) increase the computation time of both methods and decrease the network size at which the SMC-EEM method becomes more computationally efficient than the standard-EEM method. (TIF) [file pcbi.1011976.s001.tif]

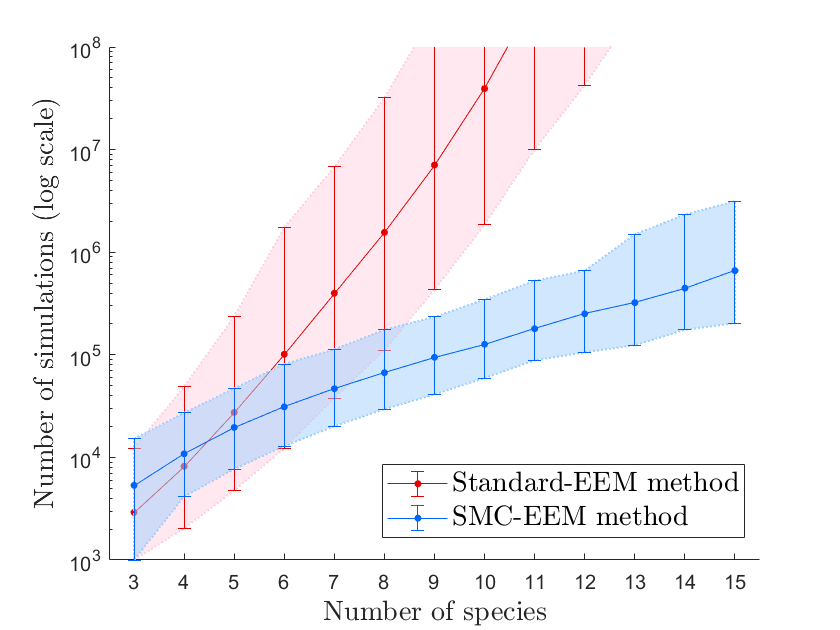

Supplement: S2 Fig — The number of parameter sets trialled to generate an ensemble of 1000 feasible and stable ecosystem models using both the standard-EEM and SMC-EEM parameterisation methods. This figure shows the medians (dots) and 7.5–92.5% quantiles (error bars) of simulation numbers for the models parameterised in Fig 2 of the manuscript. Note, the computation time for any one ecosystem network was capped at 104 seconds due to the computational burden of the simulation study. (TIF) [file pcbi.1011976.s002.tif]

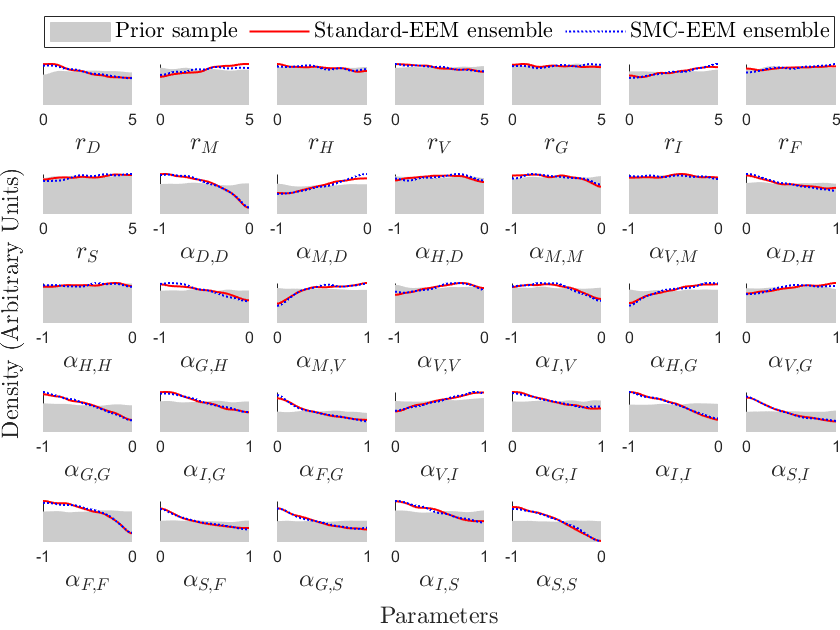

Supplement: S3 Fig — Marginal parameter distributions estimated using both the standard-EEM method (red) and the SMC-EEM method (blue). Species labels represent dingoes (D), mesopredators (M), large herbivores (H), small vertebrates (V), grasses (G), invertebrates (I), fires (F) and soil quality (S). Notice that the blue and red densities match almost exactly, demonstrating that the outputs of the standard-EEM and SMC-EEM methods are consistent. (TIF) [file pcbi.1011976.s003.tif]

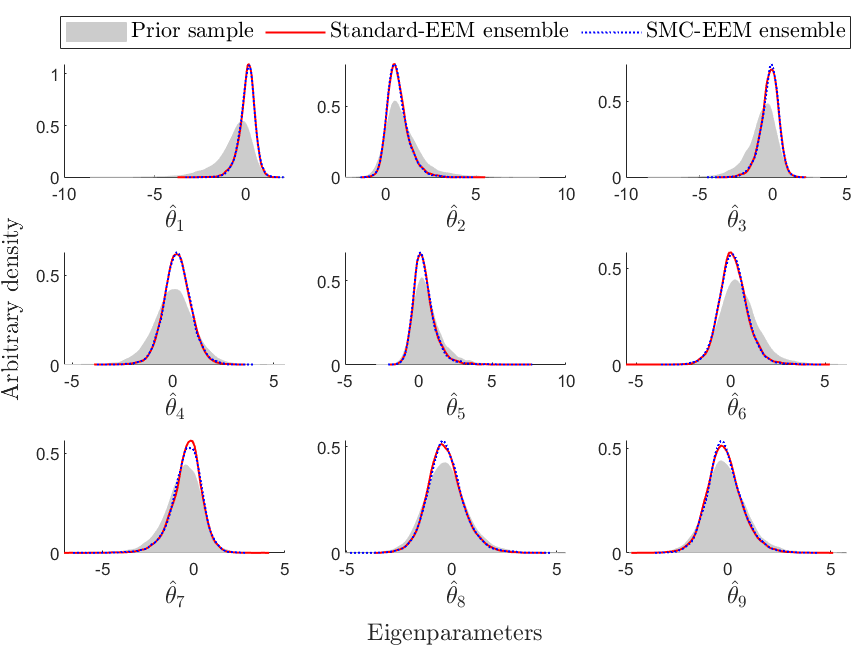

Supplement: S4 Fig — Marginal distributions of the nine stiffest eigenparameters estimated via the prior (grey), standard-EEM (red) and SMC-EEM (blue) ensembles. Notice that the blue and red densities match almost exactly, demonstrating that the outputs of the standard-EEM and SMC-EEM methods are consistent. (TIF) [file pcbi.1011976.s004.tif]

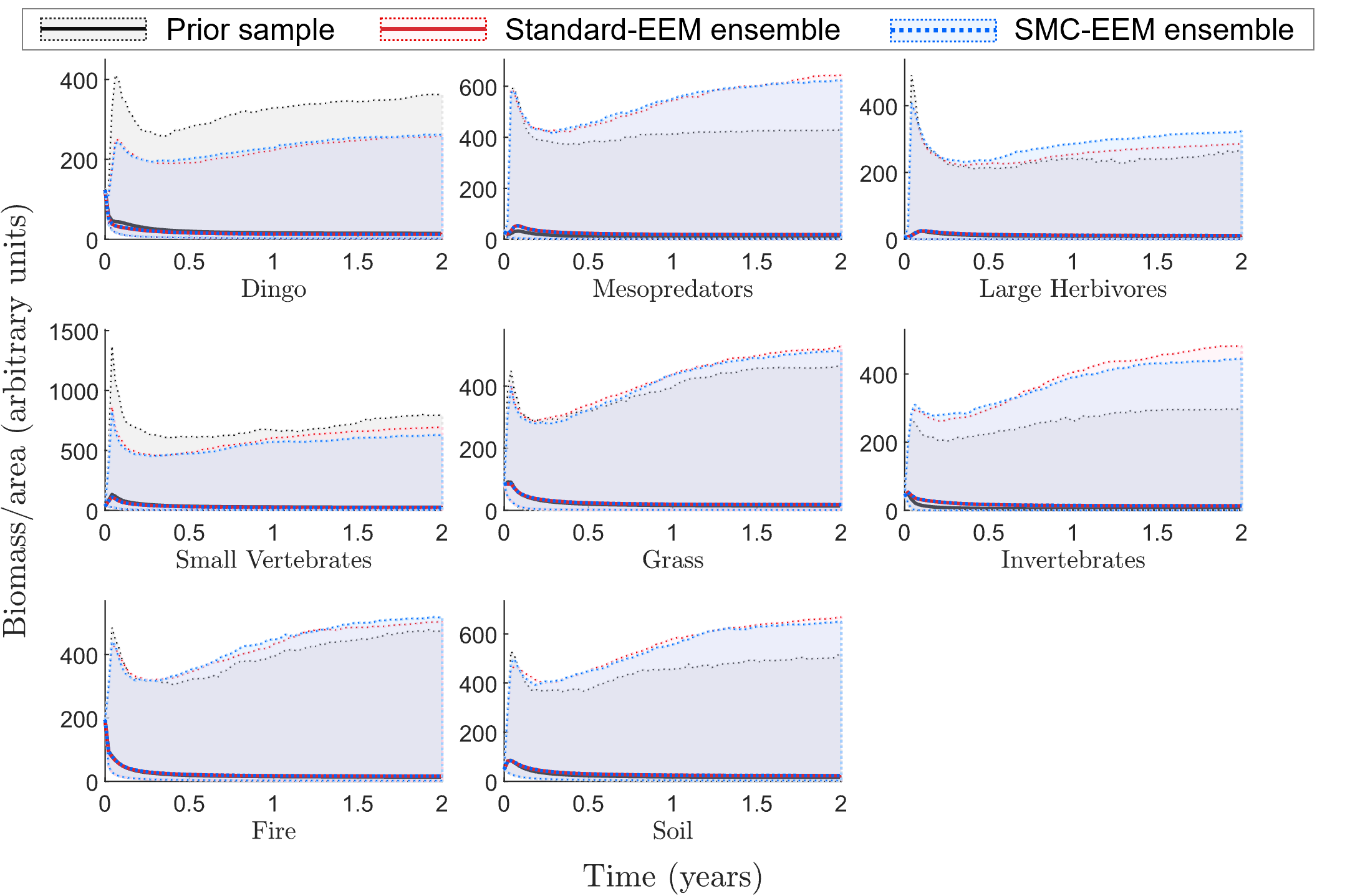

Supplement: S5 Fig — Time-series forecasts for the prior (grey), standard-EEM (red) and SMC-EEM (blue) ensembles simulated from a random initial condition. Depicted are the median (think lines) and 95% credible intervals (thin dotted lines) for each ensemble. Notice that the blue and red predictions are similar, demonstrating that the outputs of the standard-EEM and SMC-EEM methods are consistent. (TIF) [file pcbi.1011976.s005.tif]

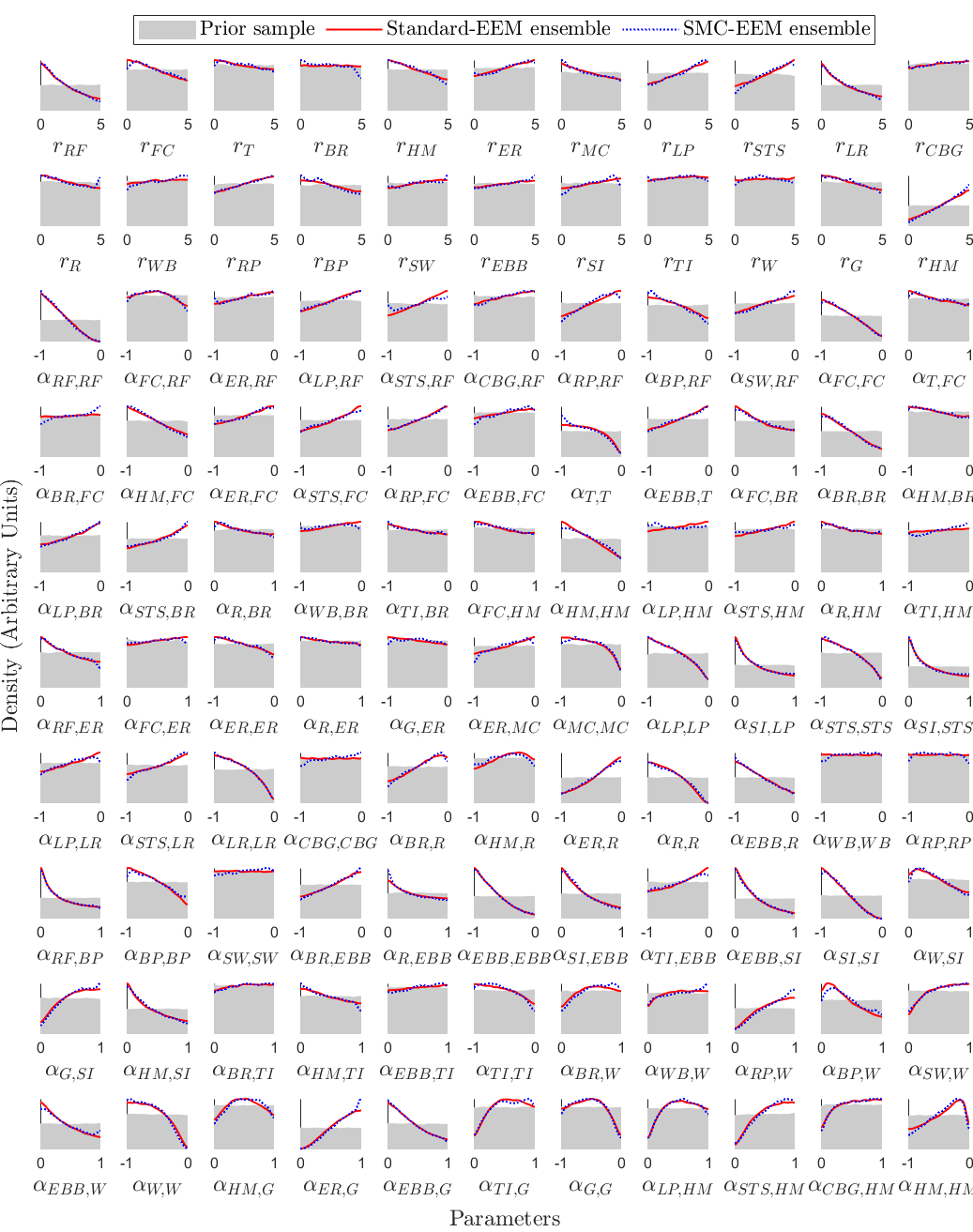

Supplement: S6 Fig — The estimated marginal distributions for each parameter within the ecosystem model for the Phillip Island network were generated via the standard-EEM method (red) and the SMC-EEM method (blue). Species labels represent parameters for the red fox (RF), feral cat (FC), toxoplasmosis (T), black rat (BR), house mouse (HM), European rabbit (ER), myxoma and calici (MC), little penguin (LP), short-tailed shearwater (STS), little raven (LR), Cape Barren geese (CBG), raptors (R), woodland birds (WB), ringtail possum (RP), brushtail possum (BP), swamp wallaby (SW), eastern barred bandicoot (EBB), soil invertebrates (SI), terrestrial invertebrates (TI), woodlands (W), grasslands (G), and herbfield (H). (TIF) [file pcbi.1011976.s006.tif]

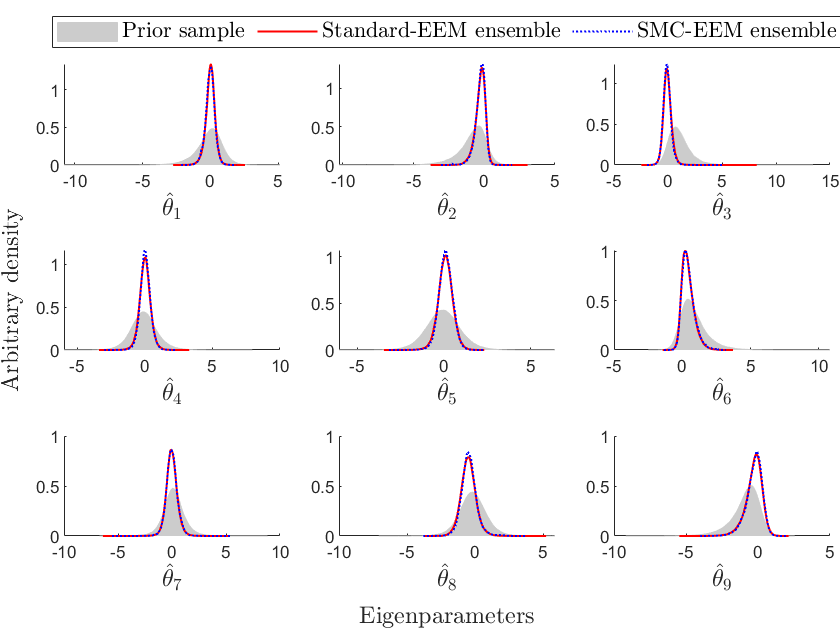

Supplement: S7 Fig — Distributions of the nine most constrained parameter combinations (stiffest eigenparameters) determined by an analysis of model sloppiness of the standard-EEM ensemble. Here we compare the values of the eigenparameters for the prior (grey), standard-EEM (red) and SMC-EEM (blue) ensemble. (TIF) [file pcbi.1011976.s007.tif]

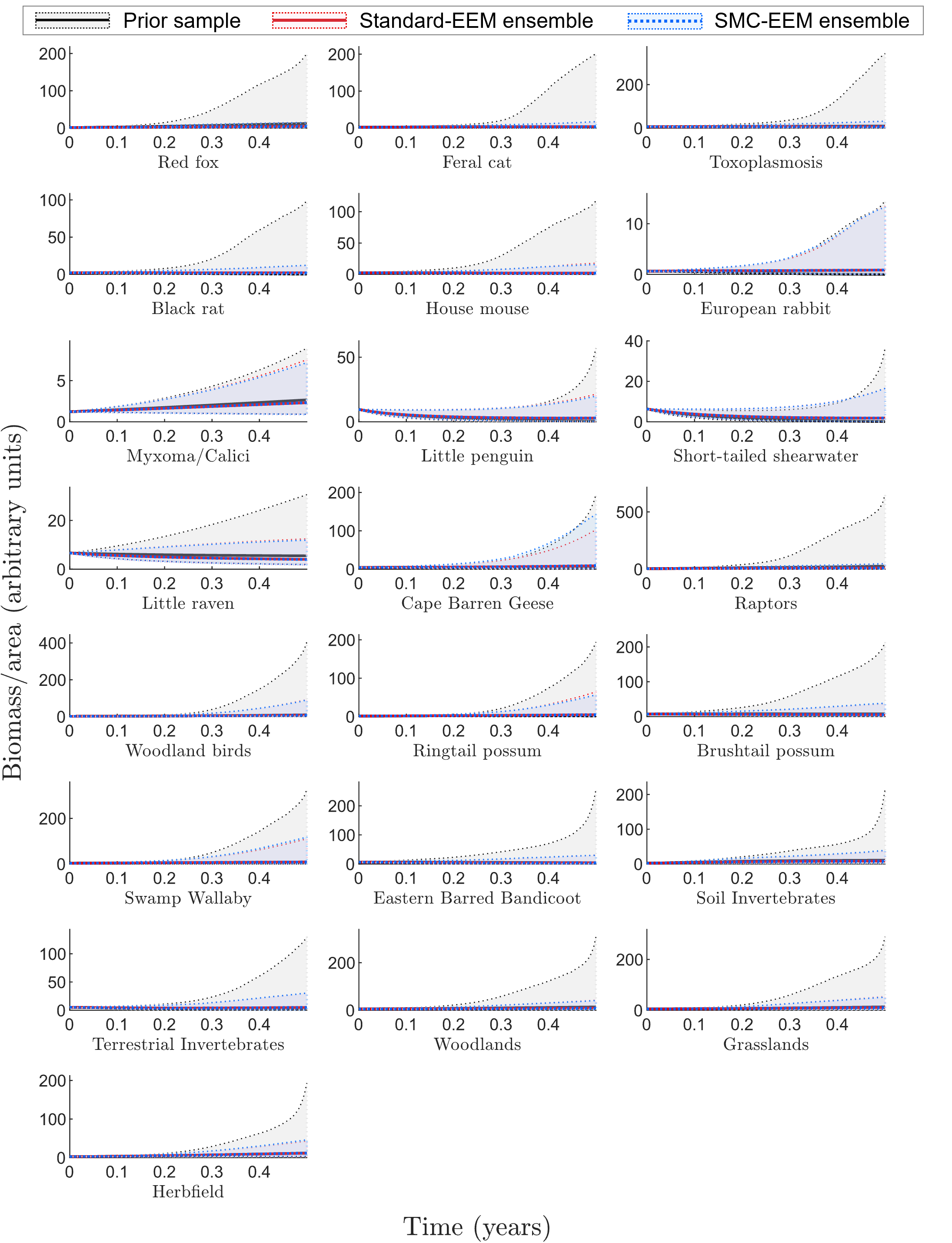

Supplement: S8 Fig — Time-series forecasts for the prior (grey), standard-EEM (red) and SMC-EEM (blue) ensembles simulated from a random initial condition. Depicted are the median (think lines) and 95% credible intervals (thin dotted lines) for each ensemble. Notice that the blue and red predictions are similar, demonstrating that the outputs of the standard-EEM and SMC-EEM methods are consistent. (TIF) [file pcbi.1011976.s008.tif]

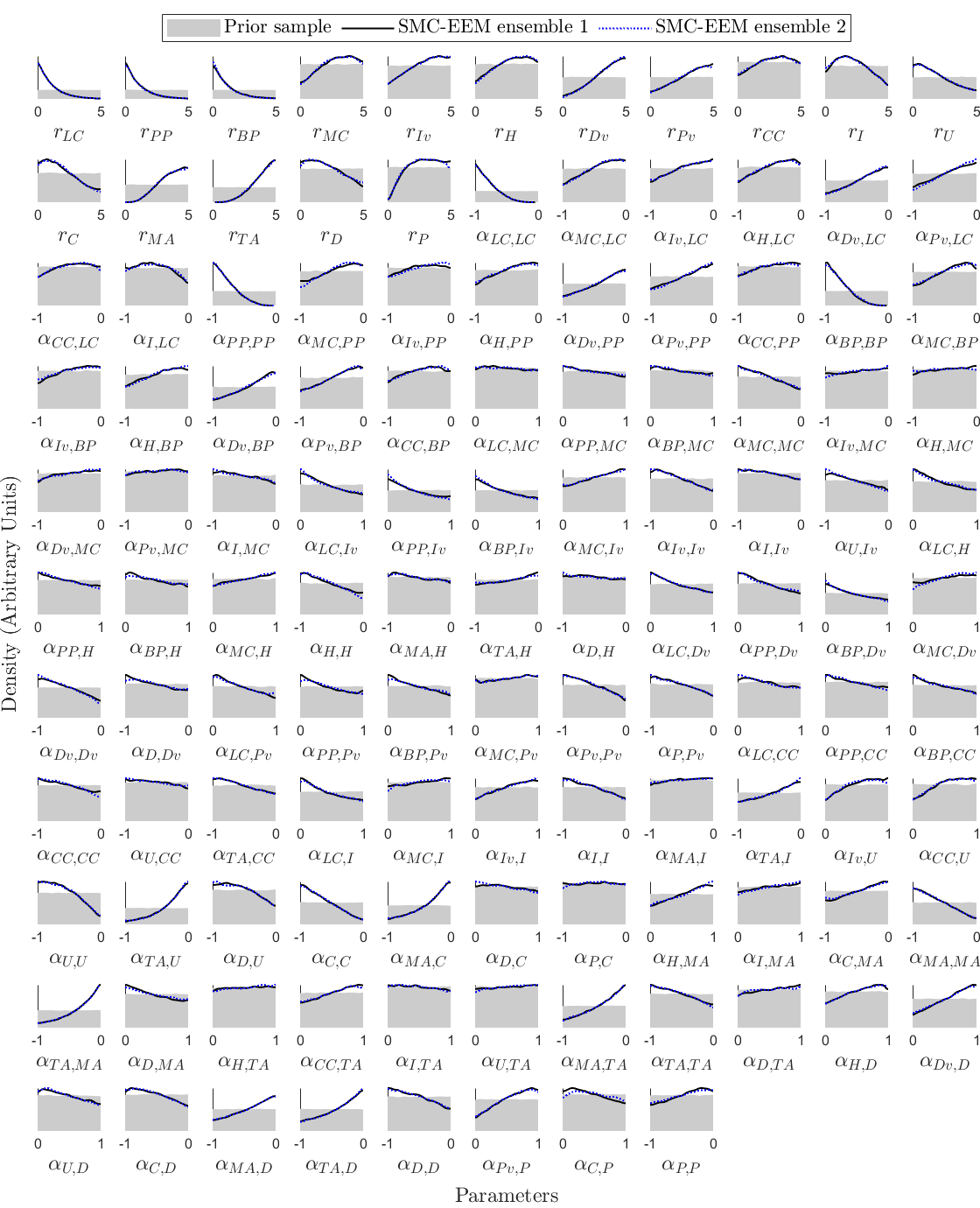

Supplement: S9 Fig — The estimated marginal distributions for each parameter within the ecosystem model for the Great Barrier Reef network were generated via two independent runs of the SMC-EEM algorithm (black and blue). Species labels represent parameters for large carnivores (LC), pelagic piscivores (PP), benthic piscivores (BP), meso-carnivores (MC), invertivores (Iv), herbivore (H), detritivores (Dv), planktivores (Pv), coral cryptics (CC), invertebrates (I), urchins (U), corals (C), macroalgae (MA), turf algae (TA), detritus (D), and plankton (P). (TIF) [file pcbi.1011976.s009.tif]

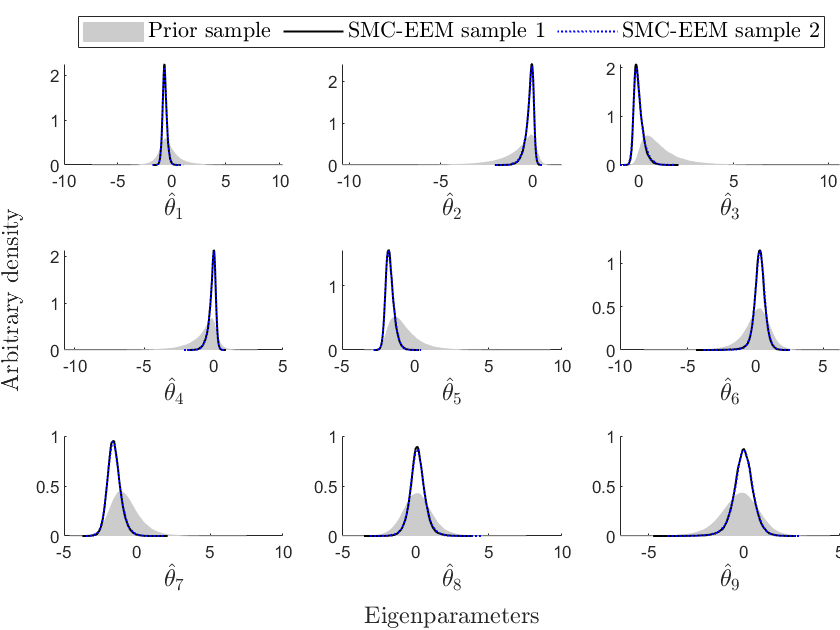

Supplement: S10 Fig — Distributions of the nine most constrained parameter combinations (stiffest eigenparameters) determined by an analysis of model sloppiness of a SMC-EEM ensemble. Here we compare the values of the eigenparameters for the prior distribution (grey), and two independent ensembles generated via the SMC-EEM algorithm (black and blue). (TIF) [file pcbi.1011976.s010.tif]

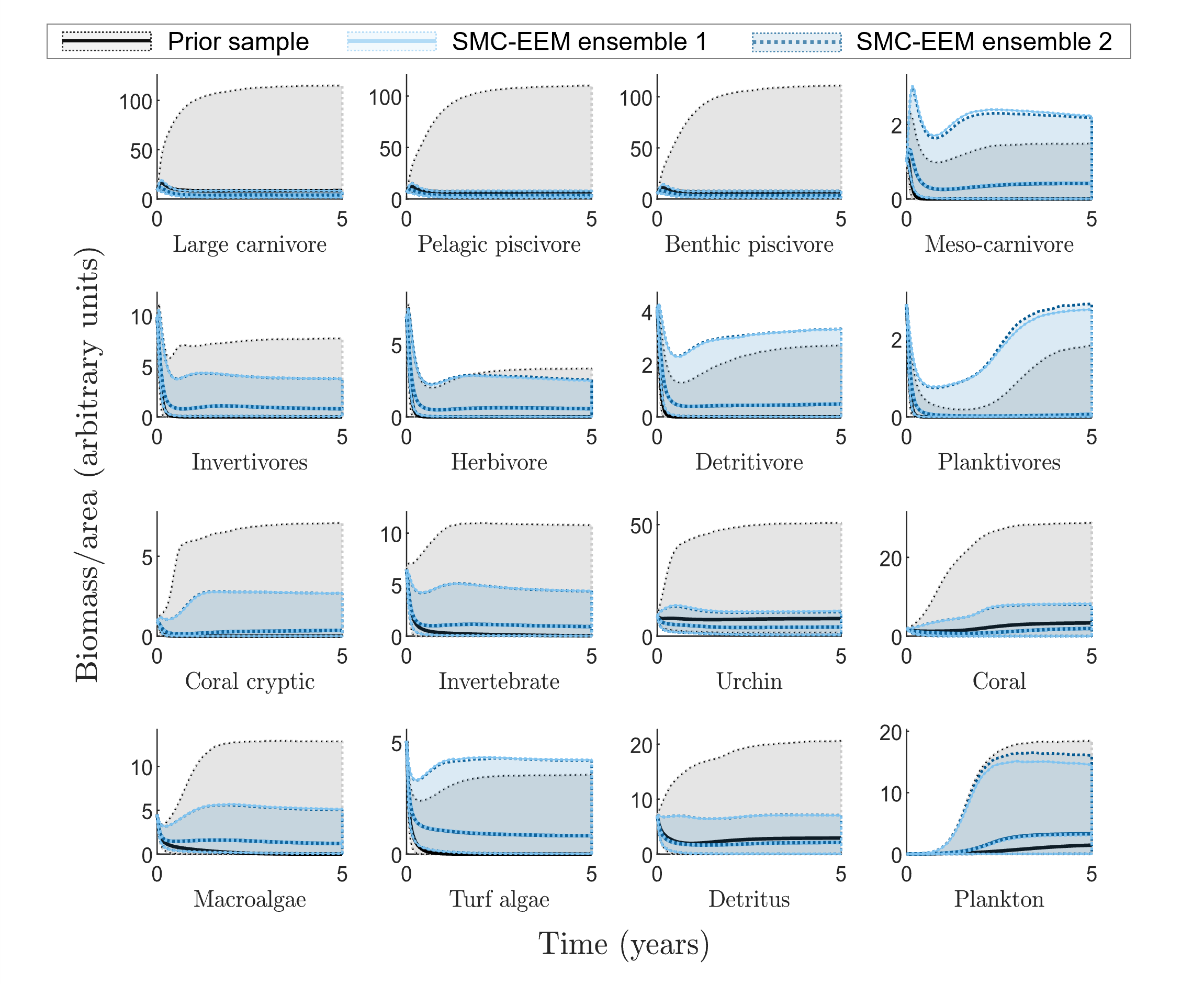

Supplement: S11 Fig — Time-series forecasts for the prior (grey), and two independently generated SMC-EEM (light and dark blue) ensembles simulated from a random initial condition. Depicted are the median (think lines) and 95% credible intervals (thin dotted lines) for each ensemble. Notice that the two blue predictions are similar, demonstrating that the SMC-EEM ensembles are consistent. (TIF) [file pcbi.1011976.s011.tif]

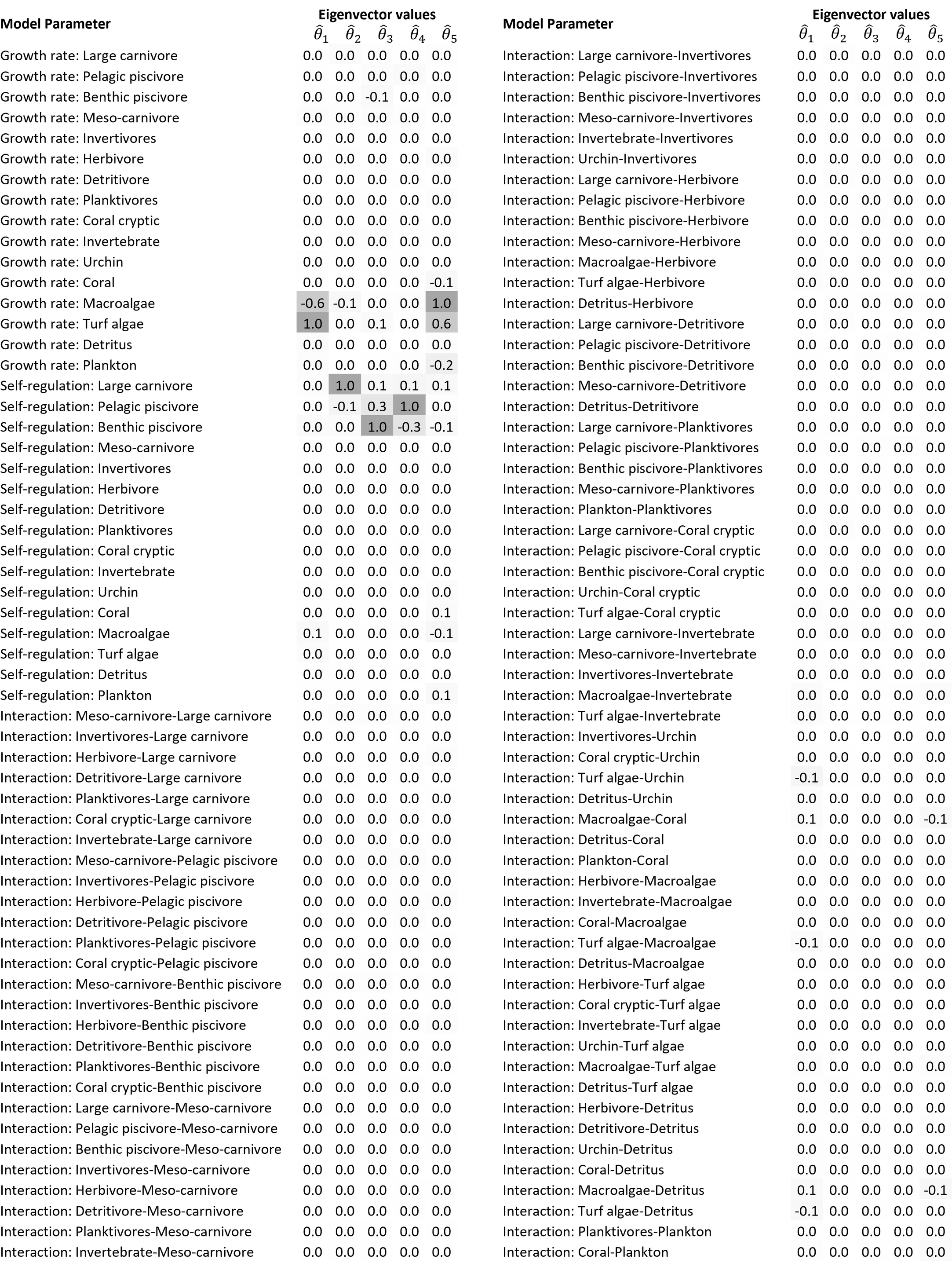

Supplement: S12 Fig — The eigenvector values for the first five eigenparameters, rescaled to be between -1 and 1. These values are shaded such that the darker colours indicates a greater contribution of the parameter to the important parameter combinations. The columns of this table can be interpreted using Eq (10). Notice, that the most important parameters are all growth rates for lower trophic species, and self-regulation for top predators. (TIF) [file pcbi.1011976.s012.tif]

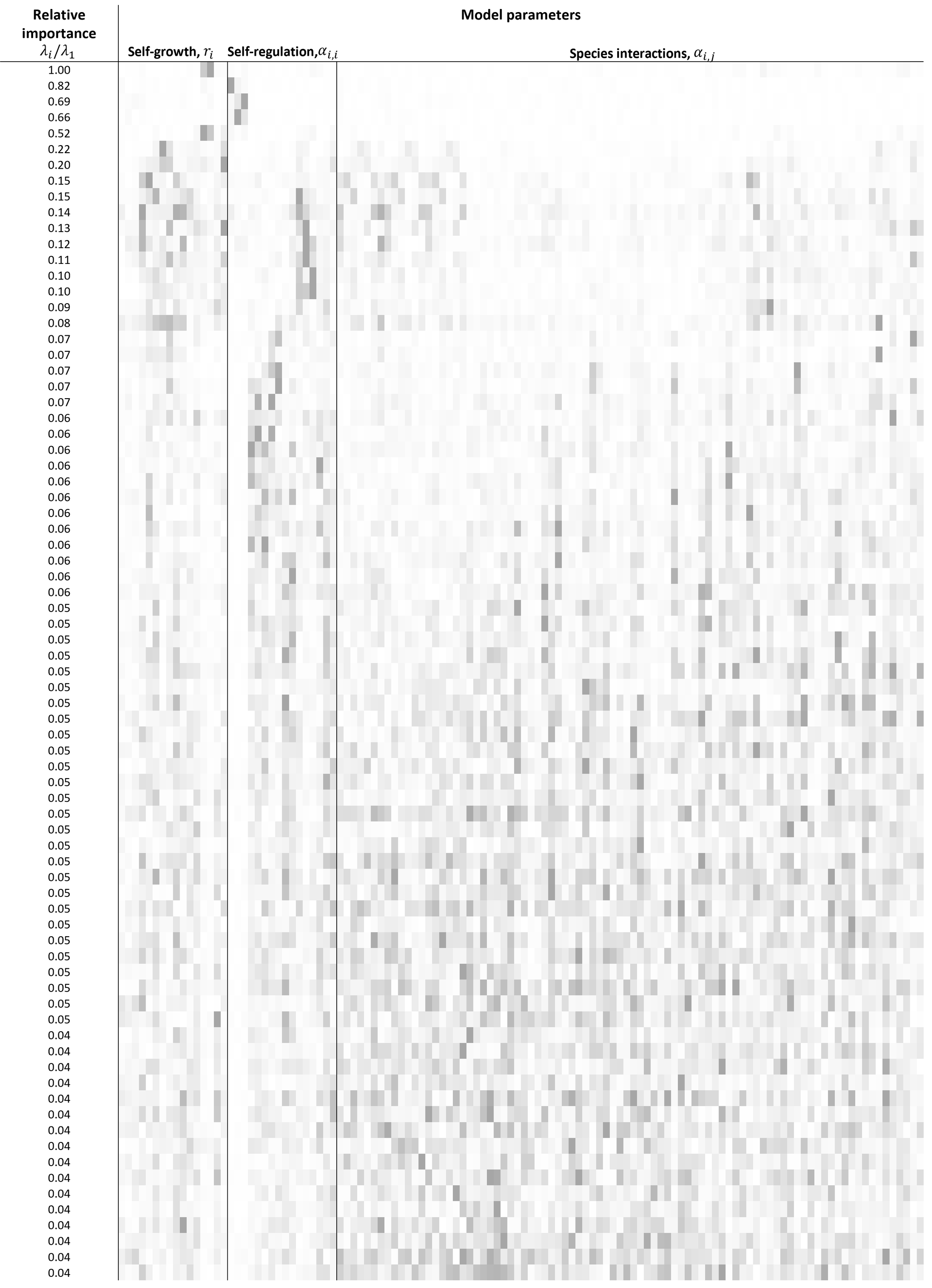

Supplement: S13 Fig — The eigenvector values for the first 80 eigenparameters, shaded such that darker colours indicate a greater contribution of the parameter to the eigenparameter. Each row represents an eigenparameter (ordered from most sensitive to least) and each column represents a model parameter (grouped by type). Note that beyond the first five eigenparameters, there are no clearly interpretable trends. (TIF) [file pcbi.1011976.s013.tif]
