# Supplementary material for: Unlocking ensemble ecosystem modelling for large and complex networks
Source: PLoS Comput Biol. 2024 Mar 14;20(3):e1011976. doi: 10.1371/journal.pcbi.1011976 (PMC10965070; doi:10.1371/journal.pcbi.1011976)
Supplement: S1 File — Additional details for implementing the SMC-EEM method, adapted from Drovandi and Pettitt’s (2011) [33] implementation of SMC-ABC. (PDF) [file pcbi.1011976.s014.pdf]

S1 File. Unlocking ensemble ecosystem modelling for large and complex networks

Sarah A. Vollert[a,b,*], Christopher Drovandi[a,b], & Matthew P. Adams[a,b,c]
[a]Centre for Data Science, Queensland University of Technology, Brisbane, Australia
[b]School of Mathematical Sciences, Queensland University of Technology, Brisbane, Australia
[c]School of Chemical Engineering, The University of Queensland, St Lucia, Australia
[*]Corresponding author. E-mail: sarah.vollert@hdr.qut.edu.au

Additional details of the SMC-EEM method

Algorithm 2 of the manuscript provides an overview of the SMC-EEM method. Here, we provide additional details for implementing the method (Algorithms S1 and S2), adapted from Drovandi and Pettitt's implementation of SMC-ABC [1]. Algorithm S1 describes the SMC-ABC algorithm including reweighting, resampling and moving the particles. Algorithm S2 details the MCMC-ABC process used within the move step of Algorithm S1.

A key difference between the algorithm presented in the manuscript (Algorithm 2) and this more detailed version (Algorithms S1 and S2) is the inclusion of a bijective transform to the parameters to ensure that the reparameterisation is free from any constraints on the original parameters [1], such as the bounds of a uniform prior distribution (e.g. see Section S.4.2 of [2]). The bijective transform ensures that any MCMC-ABC proposal will respect the constraints on the original parameters. The SMC-ABC samples for the original parameterisation can be easily obtained by applying the inverse transform to each SMC-ABC sample at the end of the algorithm. The implied prior distribution in the transformed space can be calculated via a transformation of random variables.

Algorithm S1: SMC-EEM algorithm used for sampling the ensemble of feasible and stable ecosystem models.

INITIALISE

Define the discrepancy function, $\rho(\boldsymbol{\theta})$
Specify the prior distribution, $\pi(\boldsymbol{\theta})$
Select the tuning variables, including:
  The number of particles to be sampled, M
  The percentage of particles retained in each sequential step, a
  The desired probability of particles unmoved during MCMC-ABC, c
  The number of trial MCMC-ABC steps to gauge acceptance rate, n_{MCMC}
Generate a sample of M particles ($\{\boldsymbol{\theta}_i\}_{i=1}^{M}$) from the prior distribution, $\pi(\boldsymbol{\theta})$

REWEIGHT

Evaluate the discrepancy for all particles $\boldsymbol{\rho} = \{\rho(\boldsymbol{\theta}_i)\}_{i=1}^{M}$
Sort the particles $\boldsymbol{\theta}$ in ascending order of their corresponding discrepancy $\boldsymbol{\rho}$
Set the discrepancy threshold ϵ_t based on the number of particles to be retained $n_{\text{keep}} = \text{floor}(a \times M)$

while *there are infeasible or unstable models in the ensemble,* $\max(\boldsymbol{\rho}) > 0$ **do**

  RESAMPLE

  Transform current values of $\boldsymbol{\theta}$ to $\tilde{\boldsymbol{\theta}}$ such that the parameter-space is less restricted
  Duplicate retained particle values based on discrepancy $\boldsymbol{\rho}$ to replace those with $\rho_i > \epsilon_t$
  Calculate the sample covariance matrix, $\Sigma = cov(\{\boldsymbol{\theta}_i\}_{i=1}^{n_{\text{keep}}})$

  MOVE

  for *each of the* n_{MCMC} *trial MCMC-ABC steps* **do**
    Move the particles using MCMC-ABC (Algorithm S2)
  Estimate the MCMC-ABC acceptance rate for iteration t, a_t
  Determine the number of MCMC-ABC iterations to perform, $R_t = \lceil \log(c)/\log(1-a_t) \rceil$ and update $n_{MCMC} = R_t/2$
  for *each of the remaining MCMC-ABC steps,* $R_t - n_{\text{MCMC}}$ **do**
    Move the particles using MCMC-ABC (Algorithm S2)

  REWEIGHT

  Sort the particles $\boldsymbol{\theta}$ in ascending order of their corresponding discrepancy $\boldsymbol{\rho}$
  Set the discrepancy threshold based on the number of particles to be retained, $\epsilon_t = \rho(\boldsymbol{\theta}_{n_{\text{keep}}})$
  if *we are dropping feasible and stable particles,* $\rho(\boldsymbol{\theta}_i) = 0$ *where* $i > n_{\text{keep}}$ **then**
    Adjust n_{keep} to retain all feasible and stable particles where $\rho(\boldsymbol{\theta}_i) = 0$
  Ensure all $\tilde{\boldsymbol{\theta}}$ are transformed back to $\boldsymbol{\theta}$

Algorithm S2: MCMC-ABC algorithm used within the SMC-EEM (Algorithm S1)

for *each particle* i *in* $\{\boldsymbol{\theta}_i\}_{i=n_{\text{keep}}}^{M}$ **do**
  Propose a new set of parameter values $\tilde{\boldsymbol{\theta}}_i^*$ using a multivariate normal proposal distribution, $\tilde{\boldsymbol{\theta}}_i^* \sim N(\tilde{\boldsymbol{\theta}}_i, \Sigma)$
  Calculate the prior probability ($\pi(\tilde{\boldsymbol{\theta}})$ for the transform space) of the current and proposed parameter values ($\tilde{\boldsymbol{\theta}}_i$ and $\tilde{\boldsymbol{\theta}}_i^*$)
  Transform the current and proposed parameter values ($\tilde{\boldsymbol{\theta}}_i$ and $\tilde{\boldsymbol{\theta}}_i^*$) in terms of $\boldsymbol{\theta}$
  Evaluate the discrepancy $\rho(\boldsymbol{\theta}_i^*)$
  Accept or reject a particle based on a Metropolis-Hastings acceptance probability $\alpha = \min\left(1, \pi(\tilde{\boldsymbol{\theta}}_i^*)/\pi(\tilde{\boldsymbol{\theta}}_i)\right)$, if within the discrepancy threshold, $\rho(\boldsymbol{\theta}_i^*) \leq \epsilon_t$

Practitioners should note that within this algorithm there are three tuning parameters to be selected, whose values can have substantial impact on computation time and posterior samples if poorly chosen. These tuning parameters, their potential effects, and our suggested values are outlined in Table S1.

**Parameter**	**Tuning effect**	**Ensemble quality / efficiency tradeoff**	**Value used**
$\boldsymbol{a}$: the percentage of particles retained during each iteration	Retaining more particles means that some higher discrepancy particles are retained and the space around these parameter sets are explored via MCMC.	Higher values of a leads to quality ensembles (more parameter space explored), but slower computation times (more iterations needed to remove high-discrepancy particles).	40%
$\boldsymbol{c}$: the desired probability of particles unmoved during MCMC-ABC	Lower probabilities of unmoved particles decreases the number of duplicate particles retained during each iteration.	Higher values of c leads to poorer quality ensembles (more duplicate parameter sets), but faster computation times (less time needed to remove duplicates).	1%
$\boldsymbol{n_{MCMC}}$: the number of trial MCMC-ABC steps used to estimate the MCMC-ABC acceptance rate for each iteration	Once the acceptance rate is estimated, this is used to calculate how many MCMC-ABC steps will be needed to obtain the desired probability of duplicate parameters (c).	Higher values of n_{MCMC} leads to quality ensembles (accurate estimates of acceptance rate can avoid excess duplicate particles), but slower computation times (more MCMC steps than necessary used to estimate the acceptance rate).	10

Table S1: Tuning parameters of the SMC-ABC algorithm.

These parameters can be tuned to balance the trade-off between representative samples and computational efficiency. However, we suggest using the values we have recommended here and assessing the reproducibility of the posterior sample via multiple independent algorithm runs. We note that our tuning parameter choices could be considered conservative, as they favour high sample diversity over a faster and more aggressive algorithm.

S1 File References

[1] Drovandi CC, Pettitt AN. Estimation of parameters for macroparasite population evolution using approximate Bayesian computation. Biometrics. 2011;67(1):225-33.

[2] Vollert SA, Drovandi C, Monsalve-Bravo GM, Adams MP. Strategic model reduction by analysing model sloppiness: A case study in coral calcification. Environmental Modelling & Software. 2023;159:105578.
